# Complete genome sequences of three avian pathogenic *Escherichia coli* strains isolated from colibacillosis-affected poultry in Pakistan

**DOI:** 10.1128/mra.00307-25

**Published:** 2025-07-21

**Authors:** Jing Zhang, Jeremy D. R. Cherbuin, Muhammad Khan, Hira Niaz, Fazal Adnan

**Affiliations:** 1 Department of Infectious Diseases and Pathobiology, Vetsuisse Faculty, Institute of Veterinary Bacteriology, University of Bern, Bern, Switzerland; 2 Graduate School for Cellular and Biomedical Sciences, University of Bern 27210, Bern, Switzerland; 3 Multidisciplinary Center for Infectious Diseases (MCID), University of Bern 27210, Bern, Switzerland; 4 Atta ur Rahman School of Applied Biosciences (ASAB), National University of Sciences and Technology (NUST) 66959 , Islamabad, Pakistan; The University of Arizona, Tucson, Arizona, USA

**Keywords:** colibacillosis, avian pathogenic *Escherichia coli*, PacBio sequencing

## Abstract

Colibacillosis, caused by avian pathogenic *Escherichia coli* (APEC), affects the poultry industry worldwide. APEC infections impact food supply, especially in low- and middle-income countries, and more knowledge on APEC diversity will foster research towards better control measures. Here, we present the complete genomes of three APEC strains isolated in Pakistan.

## ANNOUNCEMENT

Avian pathogenic *Escherichia coli* (APEC), a subset of extraintestinal pathogenic *E. coli*, causes colibacillosis in poultry, leading to substantial economic losses (1, 2). Despite their importance, the genetic determinants of APEC virulence and pathogenicity remain poorly understood, highlighting the need for high-quality genomic sequences to advance disease research and control.

Here, we present the complete genome sequences of three APEC strains, isolated from broiler chickens on commercial poultry farms in the humid subtropical region of central Pakistan: PE53 (O109:H51), PE136 (O-:H5), and PE143 (O36:H5). These strains were isolated from liver (PE53, PE136) and spleen (PE143) tissue of 3-week-old chickens that died of systemic colibacillosis, exhibiting fibrinous hepatitis, pericarditis, perihepatitis, peritonitis, and airsacculitis. Approximately 1 g of tissue was homogenized in 10 mL peptone-buffered water and incubated overnight at 37°C. Enriched samples were streaked onto MacConkey agar, and lactose-fermenting colonies were subcultured on eosin methylene blue (EMB) agar. Colonies displaying a green metallic sheen were confirmed as *E. coli* using *uidA* PCR.

Confirmed strains were streaked on Luria-Bertani (LB) agar. Single colonies were grown overnight at 37°C and 220 rpm in 3 mL LB broth. A 0.5 mL aliquot of each culture was pelleted for genomic DNA extraction using the Wizard Genomic DNA Purification Kit (Promega). DNA quality and quantity were assessed using Fragment Analyzer (Advanced Analytical Technologies) and Qubit fluorometry (Thermo Fisher Scientific), respectively. DNA was sheared by tagmentation, and fragments <3 kb were removed using 35% diluted AMPure beads, as part of the LongPlex Long Fragment Multiplexing Kit protocol (seqWell). Libraries were prepared using the LongPlex Kit and SMRTbell Prep Kit 3.0 (Pacific Biosciences), and profiled on a Fragment Analyzer. Sequencing was performed on a PacBio Revio system (1 SMRT Cell 25M, 30 hour movie time, Revio chemistry).

**Fig 1:**
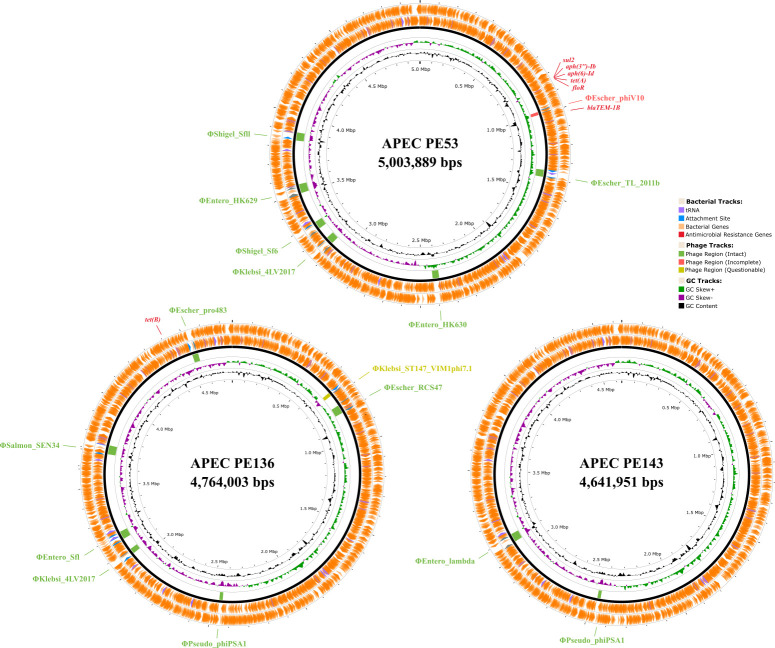
Circular genome map of APEC strains PE53, PE136, and PE143 isolated from colibacillosis-affected chicken in Pakistan. The map illustrates the genomic architecture of the APEC strains, including the distribution of GC content, the locations of antibiotic resistance genes (PE53: *sul2*, *aph(3'')-Ib*, *aph(6)-Id*, *tet(A*), *floR*, *blaTEM-1B*; PE136: *tet(B*)) along the bacterial genome, tRNA genes, intact prophage regions (PE53: ΦShigel_Sfll, ΦEntero_HK629, ΦShigel_Sf6, ΦKlebsi_4LV2017, ΦEntero_HK630, ΦEscher_TL_2011b; PE136: ΦEscher_pro483, ΦSalmon_SEN34, ΦEntero_Sfl, ΦKlebsi_4LV2017, ΦPseudo_phiPSA1, ΦEscher_RCS47; PE143: ΦEntero_lambda, ΦPseudo_phiPSA1), and putative prophage regions (PE53: ΦEscher_phiV10; PE136: ΦKlebsi_ST147_VIM1phi7.1).

Default parameters were used for all software. Adapter trimming and demultiplexing were performed using SMRT Link (v13.01), followed by additional adapter removal using LongPlex Demultiplex Nextflow (seqWell). Reads were quality-checked using NanoPlot (v1.44.1) (3). Assemblies were generated with Flye (v2.9.4-b1799) (4, 5). Assembly quality was assessed by Quast (v5.2.0) (6) and CheckM (integrated in PGAP) (7). Circular genomes were rotated to start at *dnaA* using Circlator (v1.5.5) (8), and annotated using PGAP (v2024-07-18) (9). Sequence types were determined using PubMLST (10), serotypes with SerotypeFinder (v2.0) (11), plasmids with PlasmidFinder (v2.1) (12, 13), and antimicrobial resistance genes with ResFinder (v4.6.0) (14). The final assemblies are detailed in Table 1. A circular genome map with prophage sequence prediction was generated using PHASTEST (15) (Fig. 1).

**TABLE 1:** Sequencing statistics and genomic characteristics of APEC strains PE53, PE136, and PE143 isolated from colibacillosis-affected chicken in Pakistan^*a*^

	APEC PE53				APEC PE136			APEC PE143		
Origin	Pakistan				Pakistan			Pakistan		
Specimen	Chicken liver				Chicken liver			Chicken spleen		
Total no. of reads	289,715				146,055			297,389		
Read N_50_ (bp)	8,774				7,240			7,923		
GC content (%)	50.38				50.82			50.71		
ENA accession no.	ERR14787567				ERR14787568			ERR14787569		
Assembly accession no.	GCA_965234745				GCA_965234625			GCA_965234615		
No. of contigs	4				3			3		
Assembly N_50_ (bp)	5,003,889				4,764,003			4,641,951		
Location	Chromosome	pPE53_1	pPE53_2	pPE53_3	Chromosome	pPE136_1	pPE136_2	Chromosome	pPE143_1	pPE143_2
Total size (bp)	5,003,889	168,977	220,965	60,763	4,764,003	18,819	36,004	4,641,951	124,393	126,843
Coverage (x)	421	420	337	307	204	109	187	454	197	147
No. of CDSs	4,796	185	233	74	4,593	19	39	4,386	142	135
No. of tRNA genes	87	0	0	0	87	0	0	83	0	0
Circularity	Y	Y	Y	Y	Y	Y	Y	Y	Y	Y
MLST (Achtman)	156	–^*b*^	–	–	3,871	–	–	206	–	–
Serotype	O109:H51	–	–	–	O-:H5	–	–	O36:H5	–	–
Plasmid	–	IncFIA	IncHI2	IncI2(Delta)	–		IncN	–	IncFIB(pB171)	p0111
AMR gene(s)	*aph(6)-Id, aph(3'')-Ib, blaTEM-1B, floR, sul2, tet(A*)	*aph(6)-Id, aph(3'')-Ib, blaTEM-1B, sul2, dfrA14*	*aph(4)-Ia, aph(6)-Id, aph(3')-Ia, aph(3'')-Ib, aadA1, aadA17, aadA2b, aac(3)-IV, lnu(F), cmlA1, sul3*	*mcr-1.1*	*tet(B*)		*blaTEM-1B, OqxB, OqxA, qnrS1*			*aph(6)-Id, aph(3'')-Ib, blaTEM-220, blaTEM-135, blaTEM-126, blaTEM-106, blaTEM-1B, qnrS1, tet(A), dfrA15*

^*a*^ ENA, European Nucleotide Archive; CDSs, coding DNA sequences; tRNAs, transfer RNAs; MLST, multilocus sequence typing.

^*b*^ –, not applicable.

The assembly GC contents ranged from 50.38% to 50.82%. Assemblies for PE53, PE136, and PE143 yielded 4, 3, and 3 contigs, respectively, each comprising one chromosome (4.64–5.00 Mb) and 2–3 plasmids (18.81–220.96 kb), with coverages varying between 109× and 454×. Chromosomes contained 4,386–4,914 coding sequences (CDSs) and 83–87 tRNA genes, while plasmids carried 19–233 CDSs and no tRNAs. All strains harbored antimicrobial resistance genes, with *aph(6)-Id*, *aph(3'')-Ib*, and *blaTEM-1B* being the most common. Plasmid replicons included IncFIA, IncHI2, IncI2(Delta), IncN, IncFIB(pB171), and p0111. Each genome contained 2–7 putative prophage regions (Fig. 1), suggesting the presence of integrated bacteriophages within the APEC genomes.

## Data Availability

The complete genome sequences of APEC strains PE53, PE136, and PE143 have been deposited in the European Nucleotide Archive (ENA). The assembled genomes are available under accession numbers GCA_965234745.1, GCA_965234625.1, and GCA_965234615.1, respectively. The associated BioSample are SAMEA117869410, SAMEA117869411, and SAMEA117869412, respectively. The raw PacBio HiFi sequencing reads are under accession numbers ERR14787567, ERR14787568, and ERR14787569, respectively. All data are part of BioProject PRJEB87620.
